# *Cochlospermum regium* Leaf Extract Gel: A Natural Strategy Against Methicillin-Resistant *Staphylococcus aureus*

**DOI:** 10.3390/gels11100831

**Published:** 2025-10-17

**Authors:** Fernanda Galvão, Cleison Leite, João Andrade, Pamella Castilho, Thiago Castro, Claudia Cardoso, Deisiany Ferreira, Melyssa Negri, Fabiana Dantas, Kelly Oliveira

**Affiliations:** 1 Faculdade de Ciências da Saúde, Universidade Federal da Grande Dourados (UFGD), Dourados 79804-970, Brazil; fernandagalvao@ufgd.edu.br (F.G.); cleison.leite033@academico.ufgd.edu.br (C.L.); joao.santos205@academico.ufgd.edu.br (J.A.); pamella.castilho414@academico.ufgd.edu.br (P.C.); 2 Departamento de Química, Universidade Estadual de Mato Grosso do Sul (UEMS), Dourados 79804-970, Brazil; 09611066970@academicos.uems.br (T.C.); claudia@uems.br (C.C.); 3 Departamento de Análises Clínicas, Universidade Estadual de Maringá (UEM), Maringá 87020-900, Brazil; pg55318@uem.br (D.F.); mnegri@uem.br (M.N.); 4 Faculdade de Ciências Biológicas e Ambientais, Universidade Federal da Grande Dourados (UFGD), Dourados 79804-970, Brazil; fabianasilva@ufgd.edu.br

**Keywords:** antibacterial gel, natural products, topical use, safety, ex vivo, skin infection

## Abstract

Background: Skin infections caused by *Staphylococcus aureus* represent a major public health concern, and plant extracts, such as those from *Cochlospermum regium*, have emerged as promising therapeutic alternatives. Methods: This study developed carbopol-based gel formulations containing ethanolic leaf extracts of *C. regium* (CRG 0.5% and 1%) and evaluated their physicochemical stability, antibacterial activity against *S. aureus* and a methicillin-resistant wound isolate, antioxidant potential, and biocompatibility. Results: Both CRG 0.5% and 1% were physically stable and maintained antibacterial activity for up to 90 days at 8 °C, while at 25 °C only CRG 1% retained activity throughout the evaluation period. In ex vivo pig skin assays, CRG 1% reduced methicillin-resistant *S. aureus* contamination by 99%, outperforming the conventional topical antibacterial agent (neomycin + bacitracin), which achieved 66% inhibition. The extract also exhibited high antioxidant activity without mutagenic or hemolytic effects. Although phenolic and flavonoid contents decreased over time, CRG 1% preserved adequate levels for therapeutic application. Conclusions: These findings indicate that CRG 1% has potential as a stable, safe, and effective alternative for the treatment of topical infections, particularly those caused by methicillin-resistant *S. aureus*.

## 1. Introduction

*Cochlospermum regium* (Schrank) Pilg. is a medicinal plant known for its widespread application. Belonging to the Bixaceae Kunth family, this species is characterized as a low shrub predominantly found in the Brazilian Cerrado, but also occurs in Bolivia and Paraguay [1]. Decoctions or infusions of its roots are commonly used to treat gastritis, ulcers, arthritis, inflammation, wounds, skin conditions, and uterine, ovarian, and intestinal infections [2]. However, the use of its roots leads to unsustainable extractions, contributing to the destruction of this species.

In our previous studies [3,4], we demonstrated that the ethanolic extract of *C. regium* (EECR) leaves exhibits significant antibacterial and anti-inflammatory activities, making it a feasible alternative for controlling *Staphylococcus aureus* infections. Characterized as an opportunistic microorganism, *S. aureus* is responsible for 80–90% of skin infections, including folliculitis, impetigo, cutaneous abscess, and necrotizing fasciitis [5,6,7]. Compounding this issue, resistance to topical antimicrobial agents and the emergence of methicillin-resistant *S. aureus* (MRSA) strains [8] have intensified the search for new treatment options that can act as adjuvants for treating skin infections caused by *S. aureus*.

Scientific advancements in the development of formulations using natural extracts as active ingredients have gained prominence for treating diseases and maintaining patient quality of life. The literature indicates that among topical options, gel formulations offer advantages because of their effectiveness in active ingredient release, skin absorption, spreadability, and stability, making them one of the most accepted options for patients requiring skin infection therapy [9].

Thus, considering the antibacterial potential of the ethanolic extract of *C. regium* leaves and the need for new alternatives to control skin infections caused by *S. aureus*, this study aimed to develop a gel formulation with EECR leaves and evaluate its chemical, physical, and antibacterial properties, as well as assess the antioxidant potential and biocompatibility of the ethanolic extract.

## 2. Results and Discussion

Plant extracts are rich sources of bioactive compounds that can be exploited therapeutically, contributing to the development of new pharmacological products that assist in and/or control infections and regenerative processes. Topical formulations derived from natural extracts, such as gels, offer low costs, reduced side effects, and better environmental compatibility. Numerous scientific studies have investigated potential plant species that can be used as active ingredients in these formulations. In this context, *C. regium* can be considered as a promising and innovative alternative from its previously demonstrated attributes [3,4].

**Figure 1:**
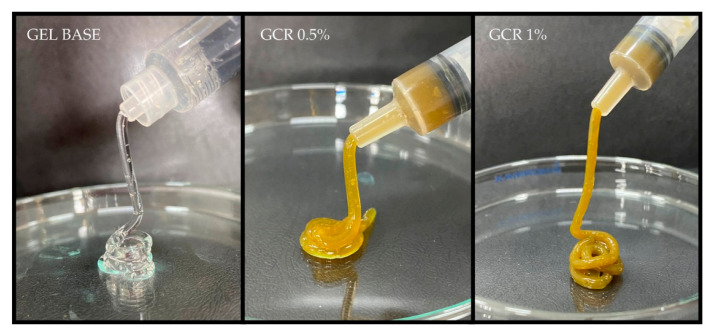
Injectability test of the developed formulations.

**Figure 2:**
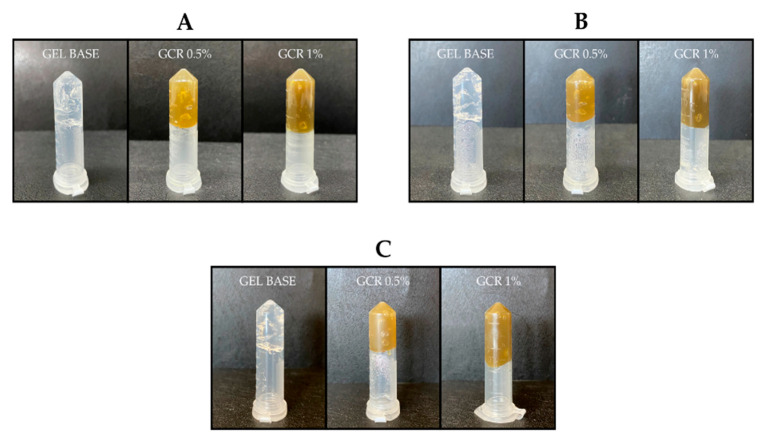
Inversion test of the developed formulations under the influence of gravity on day 0 (**A**), after 45 days (**B**), and after 90 days (**C**).

The developed gel formulations exhibited good injectability and continuous flow (Figure 1), indicating viscoelastic behavior, which allows for their use as injectable gel materials in a minimally invasive manner. Moreover, the gels demonstrated a stable shape that did not shift after tube inversion (Figure 2), even after 90 days, indicating that they did not flow under the influence of gravity. This confirms the ability of the gel to remain localized at the infection site.

The stability of the gels was evaluated under different storage conditions over 90 days by observing changes in consistency, odor, color, and signs of phase separation. Alterations in the nature of the formulations, such as precipitation, phase separation, crystallization, and lump formation, indicate instability and physical incompatibility. The formulations exhibited no significant changes, indicating their physical compatibility.

**Figure 3:**
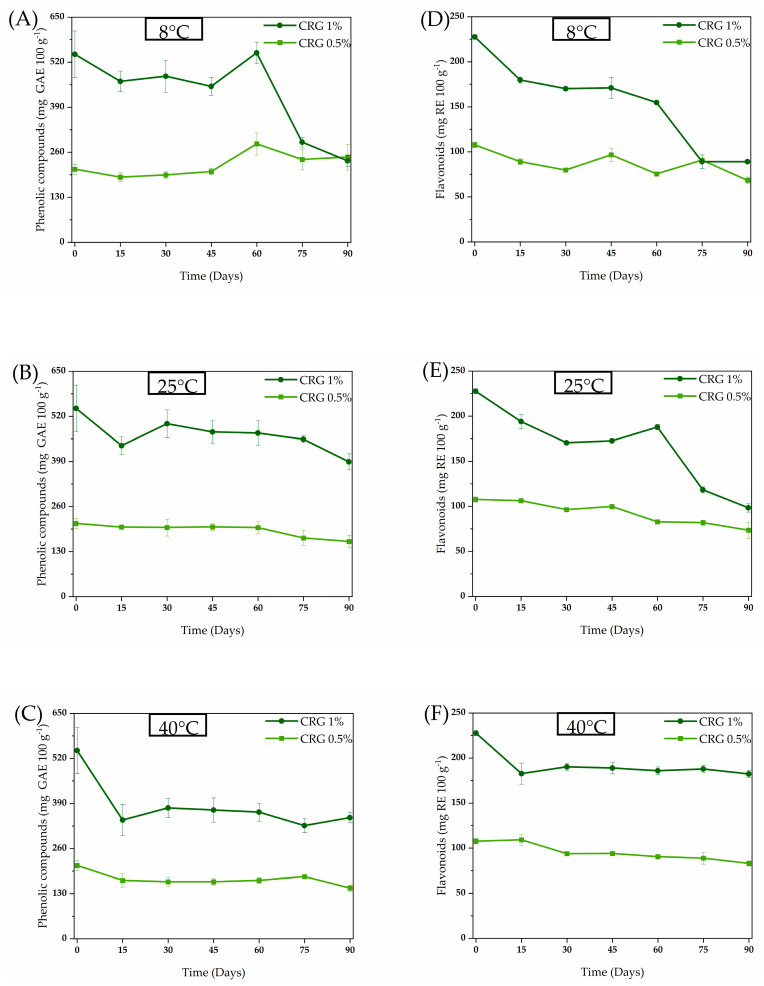
Phenolic compound content (**A**–**C**) and flavonoid content (**D**–**F**) in CRG 0.5% and CRG 1% under different storage times and conditions. CRG: *Cochlospermum regium* gel.

The chemical composition of *C. regium* has been explored previously [3,4,10]. A histochemical study conducted by Inácio et al. [10] on the central vein of *C. regium* leaves revealed the presence of phenolic compounds. Later, Galvão et al. [4] reported 25 compounds, including flavonoids, in the EECR leaves. Additionally, Galvão et al. [3] identified two phenolic compounds in the EECR leaves and associated these compounds with their effects against biofilm formation of methicillin-resistant *S. aureus*. Considering the presence of phenolic compounds in the EECR leaves and their activity against *S. aureus*, this class of compounds was monitored in the stability assay (Figure 3).

Hydrogels interact with phenolic compounds by encapsulating them within their three-dimensional network, providing protection against environmental degradation factors, such as light, oxygen, and pH variations. These factors contribute to the stabilization of compounds and preserves their bioactive properties. Furthermore, encapsulation enhances the efficacy of delivery systems in various applications [11]. However, extracts have a complex composition with many chemical interactions that may lead to the formation of other products [12,13].

Overall, all investigated treatments showed a reduction in phenolic and flavonoid content (Figure 3). The reduction was more evident with *Cochlospermum regium* gel containing 1% extract (CRG 1%), whose phenolic and flavonoid content decreased to the same level as *Cochlospermum regium* gel containing 0.5% extract (CRG 0.5%) after 90 and 75 days, respectively, when stored at 8 °C in a refrigerator. Stability may vary depending on the compound and its interactions [14,15]; high phenolic compound content may promote the formation of aggregates or reactions, further compromising formulation stability [16].

Plant metabolites often exist as soluble conjugates or insoluble bound forms. As suggested by Oliveira et al. [15] in their study on sorghum, the interplay of time and temperature can lead to the breakage of bonds between phenolic compounds and cell wall polysaccharides or other components. This hydrolysis, potentially catalyzed by residual enzymes or mild acid/base conditions in the gel, liberates aglycones and simpler phenolic molecules, which are then detected by our spectrophotometric assays. The fluctuation in compound levels on day 60 could represent a point where the rate of this release temporarily surpassed the rate of degradation.

**Figure 4:**
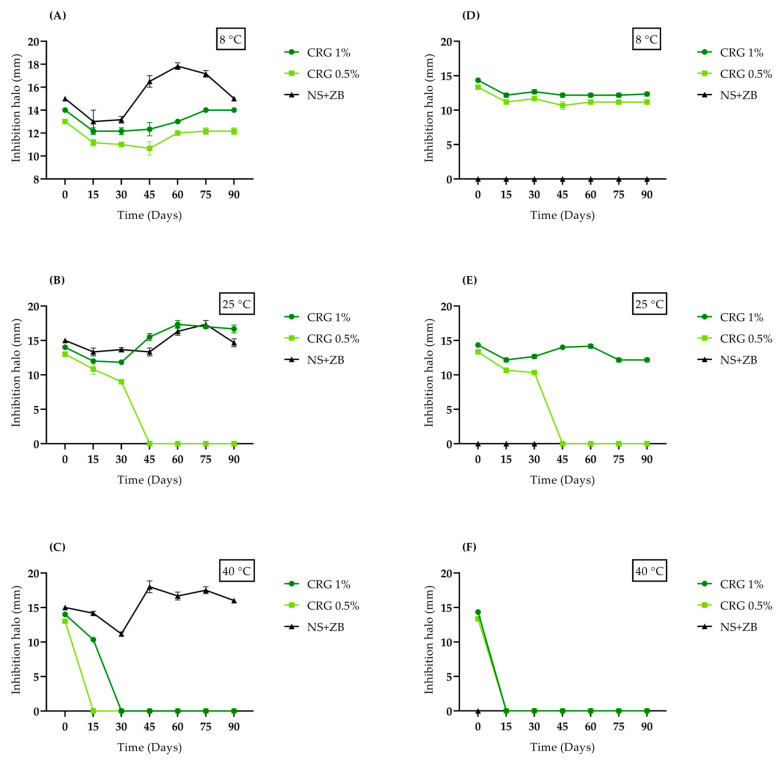
Effect of CRG 0.5% and CRG 1% at different times and storage conditions on the growth of MSSA ATCC 29213 (**A**–**C**) and MRSA (**D**–**F**). Inhibition halos expressed in mm. CRG: Gel of *Cochlospermum regium*. SN + ZB: Neomycin sulfate (5 mg/g) + Zinc bacitracin (250 µL/g). Kruskal–Wallis test (*p* < 0.0001) followed by Dunn’s post hoc test (*p* < 0.05).

At elevated temperatures, two opposing processes occur simultaneously: (1) the degradation of existing free compounds, and (2) the accelerated release of bound compounds. The results in Figure 3 suggest that, for a significant portion of the storage period, the rate of release of new detectable compounds may have counterbalanced the rate of their degradation, leading to a plateau or slower decline than anticipated [15]. However, it is important to emphasize that this chemical stability does not necessarily translate to biological stability, as the newly released compounds may have different activities,

The antibacterial activity of the *C. regium* gels at different storage temperatures over 90 days was evaluated against methicillin-sensitive *S. aureus* (MSSA) and a clinical MRSA isolate using the well-diffusion method (Figure 4). Both bacteria were sensitive to the *C. regium* gels, and the addition of 150 mg of 0.5% and 1% CRG per well initially resulted in the formation of inhibition zones of 13 and 14 mm for MSSA and MRSA, respectively. The interaction between time and temperature influenced antibacterial activity, which was enhanced at certain combinations. This may be associated with possible modifications related to the release of compounds present in the gels or their alterations. No inhibition of bacterial growth was observed when the base gel (without the EECR leaves) was added. Detailed zones of inhibition are presented in Supplementary Material S1.

**Figure 5:**
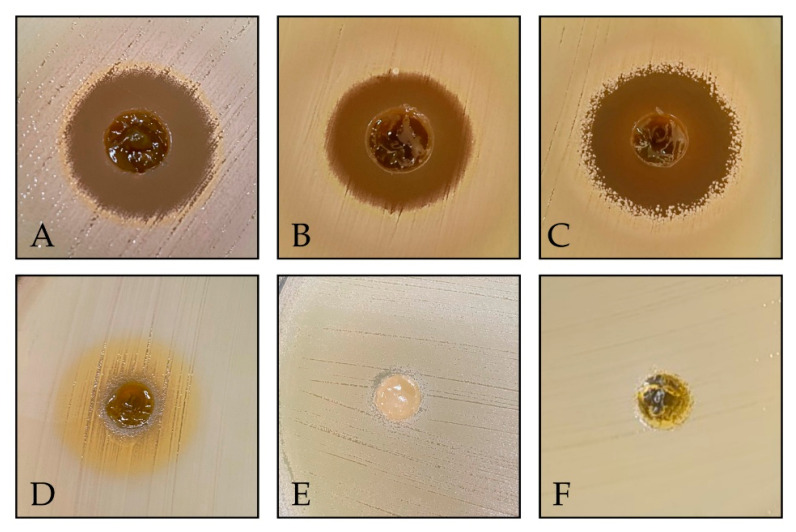
Agar well diffusion assay demonstrating the antibacterial activity of the samples against MRSA. (**A**) CRG 1% on day 0; (**B**) CRG 1% stored at 8 °C for 90 days; (**C**) CRG 1% stored at 25 °C for 90 days; (**D**) CRG 1% stored at 40 °C for 15 days; (**E**) Neomycin sulfate (5 mg/g) + Zinc bacitracin (250 µL/g); (**F**) Base Gel.

While our gel proved effective against MRSA, control of Neomycin sulfate (5 mg/g) + Zinc bacitracin (250 µL/g) (NS + ZB), a highly popular and accessible dermatological ointment for treating skin infections, showed no activity. Recently, *S. aureus* has become resistant to many topical antibacterials, including neomycin and bacitracin, which are low-cost products widely used as over-the-counter medications [8]. Our findings highlight the importance of developing natural product-based formulations to address antimicrobial resistance. Our gel demonstrated remarkable ability to evade MRSA resistance mechanisms, possibly due to the bioactive compounds present in the plant, such as flavonoids, which are a promising class of compounds against MRSA and may interfere with bacterial resistance processes (Figure 5) [17].

CRG 1% maintained its antibacterial activity for 90 days when stored at 8 °C and 25 °C. However, it lost its activity against MSSA and MRSA when stored at 40 °C after 30 and 15 days, respectively. CRG 0.5% also maintained antibacterial activity for 90 days at 8 °C. However, when stored at 25 °C and 40 °C, it lost activity after 45 and 15 days, respectively. These results indicate that the optimal storage temperature for gel stability at both concentrations is 8 °C, emphasizing the importance of keeping medications in cool environments.

Heating tends to reduce the stability of formulations because it increases the kinetic energy of the molecules, leading to more intense and frequent collisions. This increases the likelihood of chemical reactions altering the chemical composition by overcoming the activation energy barrier. This changes the entropy and enthalpy of the physicochemical system, allowing the formation of new products. Similarly, other studies have observed the negative effects of temperature on the stability of cosmetic or pharmacological formulations [18,19,20].

The observed reduction in phenolic and flavonoid content, especially at elevated temperatures (40 °C), can be attributed to accelerated degradation mechanisms such as oxidation and polymerization of these compounds. Mrázková et al. [16] demonstrated that storage at high temperatures promotes the oxidation of phenols, leading to the formation of quinones and subsequently high molecular weight polymers, which may not be detected by conventional spectrophotometric methods, yet can still influence biological activity. Furthermore, the polymerization of flavonoids may result in compounds with lower solubility and bioavailability, which could explain the loss of antimicrobial activity observed, even without a significant reduction in total phenolic content.

The stability of bioactive compounds in a semi-solid matrix is a dynamic process governed by competing chemical reactions and physical interactions. The relationship between phytochemical content and antimicrobial activity in our gel is significant but complex and non-linear. It is governed by the synergy of multiple compounds, the specific identity and potency of the molecules released from the matrix, and their progressive chemical transformation.

**Figure 6:**
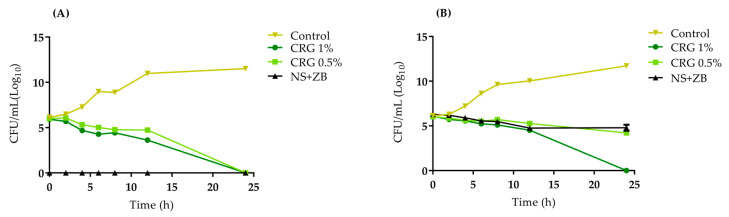
Time-kill curve of MSSA ATCC 29213 (**A**) and MRSA (**B**) in the presence of CRG 0.5% and CRG 1%. Two-way ANOVA (*p* < 0.0001) followed by Tukey’s post hoc test (*p* < 0.05).

Similar to previous antibacterial results, the kill curve profiles for MSSA and the clinical MRSA isolate at specific time intervals are presented in Figure 6. After 24 h of incubation, both gels (0.5% and 1% CRG) exhibited bactericidal effects against MSSA. Regarding MRSA, only CRG 1% demonstrated this effect, which stood out compared to the NS + ZB control, the latter having no bactericidal efficacy.

**Figure 7:**
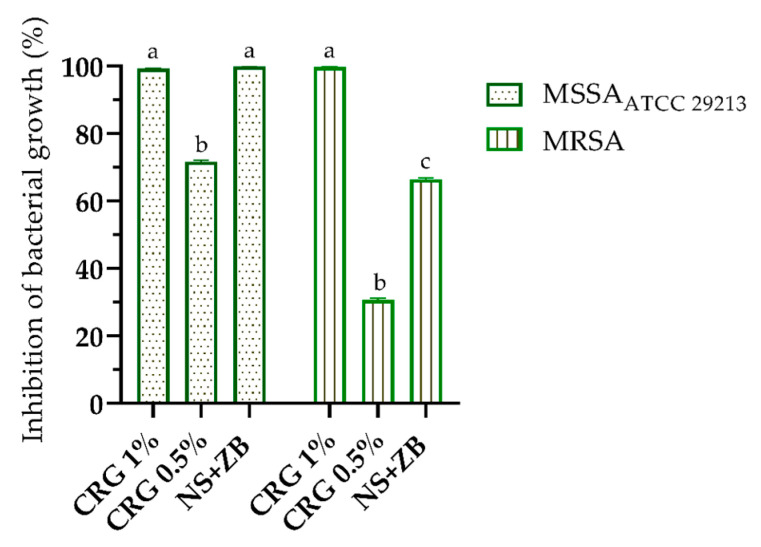
Ex vivo antibacterial activity of CRG 0.5% and CRG 1% in a pig skin model. One-way ANOVA (*p* < 0.0001) followed by Tukey’s post hoc test (*p* < 0.05) represented by the different letters.

An ex vivo model using pig skin was employed to verify the antibacterial activity of *C. regium* gels. Pig skin shares similarities with human skin, including thickness, pigmentation, the presence of hair follicles, roughness, and hydrophobicity, which facilitate microorganism adhesion [21,22]. This model is recognized for its relevance in preliminary antimicrobial evaluations; however, it does not fully reproduce the immune responses observed in living systems, warranting future in vivo investigations. The results of this assay are shown in Figure 7. The inhibition of MSSA and MRSA bacterial growth reached 99% when treated with 1% CRG in both cases. Additionally, the gel demonstrated better activity against MRSA than the NS + ZB control, the latter showing an inhibition rate of 66%. These results reinforce the potential of 1% CRG as an effective alternative for combating bacterial infections, particularly against resistant strains. When compared to other gel formulations containing natural plant extracts, evaluated through in vitro and ex vivo methods [23], the *C. regium* gel demonstrates significant potential. Its antimicrobial and biofilm-inhibiting activities position it as a competitive candidate in the development of natural topical treatments for MRSA infections. These findings highlight the efficacy of *C. regium* as a promising alternative in the field of plant-based therapies for MRSA.

To evaluate the antioxidant potential of the EECR, DPPH and ABTS free radical scavenging assays were performed. As shown in Table 1, the extract demonstrated antioxidant activity comparable to the natural antioxidant ascorbic acid, with IC_50_ values of 5.66 µg/mL in the DPPH assay and 21.81 µg/mL in the ABTS radical scavenging assay. During wound infection, an inflammatory response results in the accumulation of free radicals associated with lipid peroxidation, enzymatic inactivation, and DNA damage. Therefore, samples containing antioxidants may play an important role in the healing process [24]. The EECR showed significant anti-inflammatory and wound-healing properties, reinforcing the antioxidant potential observed in this study [4].

Given the evidence of their potential, biocompatibility assays were considered essential to confirm the safety of the gel formulations. The mutagenic potential of the *C. regium* extract is shown in Table 2. The mutagenic potential index was less than 2, and no positive dose–response relationship was observed among the concentrations. These results suggest that the EECR did not induce frameshift mutations (TA98) or base-pair substitution mutations (TA100) in the presence or absence of an exogenous metabolic activation system. Our findings are consistent with those of Nunes and Carvalho [25], who did not identify mutagenic effects of *C. regium* root extract on the germ cells of *Drosophila melanogaster*.

Another parameter evaluated was the hemolysis rate, as active compounds in bioactive formulations must exhibit high hemocompatibility, contributing to the wound healing process without causing hemotoxicity [26]. In blood samples treated with different concentrations of the *C. regium* extract, no hemolytic activity was observed, indicating the absence of cell lysis. This result indicates the hemocompatibility of EECR, considering that the in vitro cytotoxicity evaluation in mammalian cells yielded non-toxic findings.

## 3. Conclusions

Natural products based on plant extracts have gained prominence as sustainable and effective alternatives for combating bacterial infections. The results obtained herein demonstrated that 1% CRG was physically stable and compatible under different storage conditions, with the best performance at temperatures below 40 °C. The formulation was efficacious in controlling *S. aureus*, including MRSA, while exhibiting antioxidant properties and an appropriate safety profile for topical use. Thus, 1% CRG is a promising alternative for the treatment of skin infections owing to its efficacy, stability, and safety. In addition, the sustainable use of *C. regium* leaves as a raw material underscores the potential of this species as a viable industrial solution to produce natural therapeutic agents.

## 4. Materials and Methods

### 4.1. Plant Collection and Preparation of the Ethanolic Extract from Cochlospermum regium Leaves

*C. regium* leaves were collected from the Picadinha district in the municipality of Dourados, Mato Grosso do Sul. The leaves were dried in a circulating air oven at 40 °C for 96 h and ground using a knife mill (Marconi Equipamentos para Laboratórios LTDA, Piracicaba, Brazil). The dried and powdered material was extracted using sonication in two stages (45 min each) with ethanol (Merck KGaA, Darmstadt, Germany), resulting in an ethanolic extract of *C. regium* (EECR) after complete solvent removal. The plant species was identified by Dr. Zefa Valdivina Pereira, and a specimen (DDMS 5001) was deposited in the DDMS Herbarium, Biodiversity Museum, Universidade Federal da Grande Dourados (UFGD), Dourados, MS, Brazil. Research license for Brazilian biodiversity: Sistema Nacional de Gestão do Patrimônio Genético e do Conhecimento Tradicional Associado (SisGen) access registration number AA49C66.

### 4.2. Preparation of Carbopol Gel Containing Cochlospermum regium Leaf Extract

Distilled water was added to a container under a heated benchtop stirrer, followed by the addition of methylparaben (0.1%) (Merck KGaA, Darmstadt, Germany) until complete dissolution. Carbopol 940 (Merck KGaA, Darmstadt, Germany) was then added to the water with stirring until it was fully incorporated (free of lumps). Neutralization was then performed by gradually adding triethanolamine (Merck KGaA, Darmstadt, Germany) until the pH reached 6.0 to 7.0. Subsequently, different concentrations of *C. regium* leaf extract were added to the carbopol gel and mixed to prepare *C. regium* gels containing 0.5% (CRG 0.5%) and 1% (CRG 1%) of the extract.

#### 4.2.1. Evaluation of Physical Characteristics, Injectability, and Inversion Test

The stability of the formulations was evaluated under different storage conditions (8 °C, 25 °C, and 40 °C) for 90 days, and their physical characteristics, such as consistency, odor, color, and phase separation were assessed on days 0, 15, 30, 45, 60, 75, and 90. The injectability of the gel was tested by loading it into a 1 mL syringe without a needle and subjecting it to manual shear. The gel flow under the influence of gravity was observed using the inversion test, in which the *C. regium* gels were placed in 2 mL microcentrifuge tubes and kept on a flat surface without any disturbance [27].

#### 4.2.2. Quantification of Phenolic and Flavonoid Compounds

To evaluate bioactive compounds in the gel, phenolic and flavonoid compounds were quantified following the methods described by Djeridane et al. [28]. The results are expressed as milligrams of gallic acid (Merck KGaA, Darmstadt, Germany) equivalent per gram of extract (mg GAE/g) for phenolic compounds and milligrams of rutin (Merck KGaA, Darmstadt, Germany) equivalent per gram of extract (mg RE/g) for flavonoids. The gels were assessed under different storage conditions (8 °C, 25 °C, and 40 °C) on days 0, 15, 30, 45, 60, 75, and 90.

### 4.3. Antibacterial Activity

#### 4.3.1. Microorganisms

The antibacterial activities of CRG 0.5% and 1% were evaluated using a clinical isolate of methicillin-resistant *S. aureus* (MRSA) and a reference strain of methicillin-sensitive *S. aureus* (MSSA) (American Type Culture Collection, ATCC 29213) obtained from the collection of the Applied Microbiology Laboratory at the Universidade Federal da Grande Dourados.

#### 4.3.2. Agar Diffusion Test

An agar well diffusion test was performed to evaluate antibacterial activity [29]. CRG 0.5% and 1% were assessed under different storage conditions (8 °C, 25 °C, and 40 °C) on days 0, 15, 30, 45, 60, 75, and 90. The inocula were adjusted to a concentration of 1 × 10^8^ CFU/mL and seeded onto Mueller–Hinton agar (MH) (Merck KGaA, Darmstadt, Germany). Wells of 6 mm were created on agar, and 150 mg of the gel was added to the wells. The controls included the gel base and Neomycin sulfate (5 mg/g) + Zinc bacitracin (250 µL/g) (NS + ZB) (Sanofi Medley Farmacêutica, Suzano, Brazil). The plates were incubated for 24 h at 37 °C, and inhibition zones were measured and photographed.

#### 4.3.3. Time Kill

To determine the time kill curve of *S. aureus*, inoculums standardized at a concentration of 1 × 10^8^ CFU/mL were added to MH broth (Merck KGaA, Darmstadt, Germany) containing different gel concentrations (CRG 0.5% and 1%) and incubated at 37 °C. The control group was NS + ZB. Aliquots of 1.0 mL of the medium were collected at intervals of 0, 2, 4, 6, 8, 12, and 24 h, plated onto MH agar, and incubated at 37 °C for 24 h. Viable cells were counted and the results were converted to CFU/mL (Log_10_) [30].

#### 4.3.4. Ex Vivo Antibacterial Activity Assay

Fresh porcine skin (Landrace breed, aged 4–5 months) was purchased from a local butcher, thoroughly washed with sterile water, and cut into 1.5 × 1.5 cm sections. The skin was immersed in 70% ethanol for 5 min and dried in a sterile Petri dish. Each section was inoculated with 100 µL of inoculum (1 × 10^8^ CFU/mL) and incubated at 37 °C for 1 h. Subsequently, the gel was applied to each section and incubated for 4 h. The control was NS + ZB. The skin sections were then placed in 15 mL tubes containing 2 mL saline solution (Merck KGaA, Darmstadt, Germany) and agitated for 5 min. The samples were diluted and spread onto mannitol agar (Merck KGaA, Darmstadt, Germany) for colony counting, and the results are expressed as CFU/mL (Log_10_) [21].

### 4.4. Antioxidant Activity

Antioxidant activity of the EECR, tested at concentrations ranging from 50 to 5000 µg/mL, was evaluated using the 1,1-diphenyl-2-picrylhydrazyl (DPPH) (Merck KGaA, Darmstadt, Germany) free radical scavenging assay, as described by Yao et al. [31], and the 2,2′-azino-bis (3-ethylbenzothiazoline-6-sulfonic acid) (ABTS) (Merck KGaA, Darmstadt, Germany) assay, according to Xiao et al. [32]. Ascorbic acid (Merck KGaA, Darmstadt, Germany) was used as a standard antioxidant. The radical scavenging capacity was calculated using the following equation: % Inhibition DPPH/ABTS = (Abs control − Abs sample)/Abs control) × 100. The results are expressed as the concentration capable of inhibiting 50% (IC_50_) of free radicals.

### 4.5. Biocompatibility Assays

#### 4.5.1. Ames Test

The mutagenic potential of the EECR was evaluated using the Ames test with *Salmonella* Typhimurium strains TA98 and TA100 at a concentration of 1–2 × 10^9^ cell/mL, with and without the S9 fraction [33]. In each tube, 0.2 M phosphate-buffered saline (PBS) (Merck KGaA, Darmstadt, Germany) or S9 fraction (Moltox^®^, Boone, NC, USA), extract at concentrations ranging from 50 to 5000 µg/plate, and bacterial suspension were added. After pre-incubation at 37 °C for 90 min, top agar was added to the tubes, and the resulting mixture was poured onto minimal agar plates. The plates were incubated at 37 °C for 48–66 h, and revertant colonies were counted.

The mutagenicity index (MI) was calculated as the ratio between the number of induced and spontaneous revertants, with the extract was considered mutagenic when the MI was equal to or greater than 2 at any tested concentration. Distilled water was used as a negative control. Positive controls included 2-aminoanthracene (2.5 µg/plate) (Merck KGaA, Darmstadt, Germany) for both strains in assays with metabolic activation. For assays without metabolic activation, 4-nitro-o-phenylenediamine (10 µg/plate) (Merck KGaA, Darmstadt, Germany) was used for the TA98 strain, and sodium azide (2.5 µg/plate) (Merck KGaA, Darmstadt, Germany) was used for the TA100 strain.

#### 4.5.2. Hemolytic Potential

To investigate the hemolytic potential of the EECR in human erythrocytes, the study was authorized by the Research Ethics Committee for studies involving humans at the Universidade Federal da Grande Dourados (Dourados, MS, Brazil), under approval n° 5,588,196. A human blood sample was mixed with 0.9% NaCl and centrifuged to obtain erythrocytes. The centrifuged sediment was resuspended in 0.9% NaCl to obtain a 1% suspension. EECR was added to the erythrocyte suspension at concentrations ranging from 50 to 5000 µg/mL.

A suspension treated with 1% Triton-X (Merck KGaA, Darmstadt, Germany) was used as a positive control, whereas that treated with PBS served as a negative control. The samples were incubated for 1 h at 25 ± 2 °C under slow, constant agitation (100 rpm), followed by centrifugation at 1600 rpm for 10 min. Hemolysis was quantified at 540 nm [34], and the results are expressed as the concentration capable of causing 50% (IC_50_) hemolysis.

### 4.6. Statistical Analyses

The results were analyzed using analysis of variance (ANOVA). Differences were considered statistically significant at *p* < 0.05. Statistical tests and graphs were generated using GraphPad Prism software (version 9.0; GraphPad Software, San Diego, CA, USA). The results of the Ames test were analyzed using the Salanal statistical program (U.S. Environmental Protection Agency, Monitoring Systems Laboratory, USA, version 1.0, developed by the Research Triangle Institute, RTP).

## Figures and Tables

**Table 1:** IC_50_ values in µg/mL of the ethanolic extract of *C. regium* in the DPPH and ABTS assays.

Sample	DPPH	ABTS
AA	3.14 ± 0.05	4.20 ± 0.18
EECR	5.66 ± 0.07	21.81 ± 0.45

AA: Ascorbic acid; EECR: Ethanolic extract of *C. regium.*

**Table 2:** Mutagenic activity of the *C. regium* extract expressed as the mean number of revertant colonies/plate and mutagenicity index against the TA98 and TA100 strains of *Salmonella* Typhimurium in the absence (S9−) and presence (S9+) of metabolic activation.

µg/placa	TA98		TA100	
	−S9	+S9	−S9	+S9
0 ^a^	19 ± 1	19 ± 3	124 ± 11	129 ± 4
50	25 ± 1 (1.3)	16 ± 1 (0.8)	130 ± 8 (1.0)	109 ± 4 (0.8)
150	19 ± 2 (1.0)	20 ± 3 (1.0)	120 ± 4 (0.9)	108 ± 7 (0.8)
500	18 ± 1 (0.9)	23 ± 1 (1.2)	127 ± 6 (1.0)	115 ± 4 (0.8)
1500	18 ± 2 (0.9)	24 ± 3 (1.2)	128 ± 5 (1.0)	148 ± 6 (1.1)
5000	16 ± 2 (0.8)	17 ± 0.5 (0.8)	117 ± 6 (0.9)	123 ± 3 (0.9)
C+	555 ± 25 ^b^	880 ± 9 ^c^	1303 ± 7 ^d^	803 ± 8 ^c^

Negative control: ^a^ DMSO; Positive control (C+): ^b^ 4-nitrophenylenediamine (10 μg/plate); ^c^ 2 AA-aminoanthracene (1.5 µg/plate); ^d^ Sodium azide (2.5 μg/plate).

## Data Availability

The original contributions presented in this study are included in the article. Further inquiries can be directed to the corresponding author.

## Supplementary Materials

The following supporting information can be downloaded at: https://www.mdpi.com/article/10.3390/gels11100831/s1, S1: Planilha1.

## Abbreviations

The following abbreviations are used in this manuscript:

CRG 0.5%	*Cochlospermum regium* gel containing 0.5% extract
CRG 1%	*Cochlospermum regium* gel containing 1% extract
*S. aureus*	*Staphylococcus aureus*
EECR	Ethanolic extract of *Cochlospermum regium* leaves
MRSA	Methicillin-resistant *Staphylococcus aureus*
MSSA	Methicillin-sensitive *Staphylococcus aureus*
NS + ZB	Neomycin sulfate + Zinc bacitracin
IC_50_	Lowest concentration capable of causing 50% inhibition
DPPH	1,1-diphenyl-2-picrylhydrazyl
ABTS	2,2′-azino-bis(3-ethylbenzothiazoline-6-sulfonic acid)
GAE	Gallic acid equivalents
RE	Rutin equivalents
ATCC	American Type Culture Collection
CFU	Colony-forming unit
MH	Mueller–Hinton (agar/broth)
TA98	*Salmonella enterica* serovar Typhimurium strain TA98
TA100	*Salmonella enterica* serovar Typhimurium strain TA100
S9	fraction exogenous metabolic activation system
PBS	Phosphate-buffered saline
MI	Mutagenicity index
ANOVA	Analysis of variance
SisGen	Sistema Nacional de Gestão do Patrimônio Genético e do Conhecimento Tradicional Associado
